# Cognitive impairment and its associated health conditions in American Indian communities

**DOI:** 10.1038/s44400-026-00080-0

**Published:** 2026-04-29

**Authors:** Wenjun Fan, Jiahui Dai, Yuxi Shi, Erin M. Poole, Joan O’Connell, Spero M. Manson, Luohua Jiang

**Affiliations:** 1 Department of Epidemiology & Biostatistics, Joe C. Wen School of Population & Public Health, Susan and Henry Samueli College of Health Sciences, University of California, Irvine, CA USA; 2 Centers for American Indian and Alaska Native Health, Colorado School of Public Health, University of Colorado Anschutz Medical Campus, Aurora, CO USA

**Keywords:** Cognitive ageing, Public health

## Abstract

This cross-sectional study examined cognitive impairment and its associations with health conditions among American Indian (AI) adults aged ≥55 residing in the Pacific Northwest, Rocky Mountains, and Northern Plains from 2019 to 2023. Subjective cognitive impairment was assessed using the adapted AD8, a culturally tailored eight-item measure to differentiate aging and dementia. Among 712 AI adults surveyed, 34.3% had cognitive impairment, with the highest proportion observed among those aged 55–59 (40.3%). Results from negative binomial regression indicated that distress [prevalence rate ratio (PRR) = 2.25, 95% confidence interval (CI): (1.31–3.87)], head injury [PRR = 1.58, 95% CI: (1.00–2.51)], and diabetes [PRR = 1.59, 95% CI: (1.21–2.10)] were significantly associated with worse cognitive performance. Additionally, the findings suggest a high prevalence of cognitive impairment in this AI sample of convenience. Furthermore, associations between several health conditions and cognitive function differ by age groups. Early screening for cognitive impairment using tools like AD8 is recommended for AI adults.

## Introduction

Cognitive impairment is a potential early marker of Alzheimer’s disease and related dementias (ADRD) and attendant cognitive deficits^1^. A 2017–2019 study of a large, multi-tribal population-based cohort of American Indians (AIs) in the United States (U.S.) reported a prevalence of cognitive impairment of 54%, more than three times that of non-Hispanic white adults aged 70–95 years^2^. Investigating cognitive impairment within AI communities is particularly important given the rapidly growing number of older adults and their elevated risk of ADRD. They face significant challenges, including the lowest educational attainment, highest poverty rates, elevated mortality rates, and a greater burden of chronic diseases compared to other U.S. populations^3,4^. Additionally, AI adults are at risk for earlier onset of chronic diseases that increase the likelihood of cognitive decline at relatively younger age^5^. According to the 2024 *Lancet* report, several health conditions, including diabetes, depression, traumatic brain injury (TBI), hypertension, obesity, and excessive alcohol consumption, have been identified as important risk factors for ADRD, while cardiovascular disease, particularly stroke, has been identified as a precursor^6^. However, the health conditions linked to cognitive impairment among younger (i.e., <65 years) versus older (i.e., ≥65 years) AIs remain unclear. This knowledge gap underscores the need to further investigate the factors contributing to cognitive impairment in this population.

The absence of convenient, culturally sensitive assessment tools poses a major challenge to the early detection of cognitive impairment among AI, and leads to underreporting and mischaracterizing cognitive symptoms^7^. The Ascertain Dementia 8-Item Informant Questionnaire (AD8)^8^, a brief, 8-item self-report or informant-based measure, offers a promising means by which to address this limitation. Its emphasis on observable cognitive changes and ease of administration make it a strong candidate for adaptation, which we pursued to increase its cultural relevance and accuracy with this population in mind. The present study first aimed to better understand the performance of the adapted AD8 within AI communities. We then used adapted AD8 to determine the associations between various self-reported health conditions and subjective cognitive impairment (hereafter called “cognitive impairment”) among AIs across different age groups.

## Results

### Characteristics of the study sample

A total of 712 AI participants aged ≥55 years old were enrolled in the study, with a mean age of 66.1 ± 7.5 years, and a majority of them (70.4%) being female. Of these respondents, 244 (34.3%) reported two or more “Yes” responses on the AD8 questionnaire, suggesting cognitive impairment. The proportion of cognitive impairment was notably high in the 55–59 age group (40.3%), compared to 36.5%, 27.9%, in the 60–64 and 65–74 age groups, respectively (*p* < 0.05). Individuals with cognitive impairment were more likely to report distress (10.1% vs. 2.0%), diabetes (54.6% vs. 44.1%), hypertension (42.2% vs. 33.2%), head injury (17.0% vs. 7.6%), AUD (17.3% vs. 7.8%), and obesity (41.2% vs. 32.3%) (all *p* < 0.05) and had a significantly higher total number of health conditions than those without cognitive impairment (1.9 vs. 1.4, *p* < 0.001). Compared to older individuals, participants younger than 65 years had a significantly lower proportion of diabetes (38.9% vs. 55.2%, *p* < 0.001) and heart disease (14.8% vs. 22.9%, *p* < 0.01), but a higher proportion of AUD (14.6% vs. 7.8%, *p* < 0.01), head injury (13.4% vs. 8.3%, *p* < 0.05), and distress (6.5% vs. 3.2%, *p* < 0.05) [Table 1].

**Table 1:** Demographic characteristics and health conditions of American Indian participants by cognitive impairment status and age groups

	Overall	Cognitive impairment		Age groups	
		No (AD8 Score ≤ 1)	Yes (AD8 Score ≥ 2)	<65 Years	≥65 Years
Sample Size	*n* = 712	*n* = 468 (65.7%)	*n* = 244 (34.3%)	*n* = 325 (45.6%)	*n* = 387 (54.4%)
Cognitive Impairment *N* (%)	244 (34.3)	-	-	124 (38.2)	120 (31.0)^*^
Age (Mean ± SD)	66.1 ± 7.5	66.2 ± 7.1	66.0 ± 8.2	59.6 ± 3.0	**71.6** ± 5.5^***^
Age Category *N* (%)					
55–59	144 (20.2)	86 (59.7)	58 (40.3)^*^	144 (44.3)	-
60–64	181 (25.4)	115 (63.5)	66 (36.5)^*^	181 (55.7)	-
65–74	290 (40.7)	209 (72.1)	81 (27.9)^*^	-	290 (74.9)^***^
≥75	97 (13.6)	58 (59.8)	39 (40.2)^*^	-	97 (25.1)^***^
Female Sex N (%)	501 (70.4)	326 (69.7)	175 (71.7)	218 (67.1)	283 (73.1)
Health Conditions^a^					
Distress	33 (4.7)	9 (2.0)	24 (10.1)^***^	21 (6.5)	12 (3.2)^*^
Diabetes	316 (47.7)	192 (44.1)	124 (54.6)^*^	118 (38.9)	198 (55.2)^***^
Hypertension	227 (36.2)	138 (33.2)	89 (42.2)^*^	99 (34.6)	128 (37.5)
Heart Disease	117 (19.2)	70 (17.2)	47 (22.9)	42 (14.8)	75 (22.9)^*^
Stroke	42 (7.1)	27 (6.8)	15 (7.7)	20 (7.3)	22 (7.0)
Head Injury	63 (10.7)	30 (7.6)	33 (17.0)^***^	37 (13.4)	26 (8.3)^*^
Alcohol Use Disorder	66 (11.0)	31 (7.8)	35 (17.3)^***^	41 (14.6)	25 (7.8)^**^
Obesity	220 (35.3)	133 (32.3)	87 (41.2)^*^	105 (36.5)	115 (34.3)
# of health conditions (Mean ± SD)	1.5 ± 1.4	1.4 ± 1.2	**1.9** ± 1.6^***^	1.5 ± 1.4	1.6 ± 1.3

Numbers were displayed as mean and standard deviation (SD) for continuous variables; numbers were displayed as frequency (percentage) for categorical variables (row percentage for sample sizes and age categories; column percentage for all others); test of significance was conducted between those with vs. without cognitive impairment, and those younger 65 years and older 65 years; *p* < 0.05 indicates that there is a significant difference in examined covariates between status of cognitive impairment or age groups.

^*^p < 0.05, ^**^p < 0.01, ^***^p < 0.001.

^a^*n* = 3 participants have missing information on health conditions.

### Health conditions associated with the AD8 scores

Table 2 presents the results of the unadjusted and adjusted negative binomial regression analysis examining the association between health conditions and AD8 scores. In the models adjusted only for age and sex, all health conditions, except stroke, heart disease, and obesity, were significantly associated with AD8 scores. In the fully adjusted model including age, sex, and all health conditions, distress, head injury, and diabetes remained significantly associated with higher AD8 scores, although the magnitude of these associations was slightly attenuated. Specifically, distress was associated with a 2.25-fold higher AD8 score, indicative of worse cognitive function (PRR = 2.25). Head injury was associated with a 58% higher AD8 score (PRR = 1.58), and diabetes was associated with a 59% higher AD8 score (PRR = 1.59). All associations remained statistically significant (all *p* < 0.05).

**Table 2:** Association between risk factors and AD8 scores in American Indian participants using negative binomial regression

	Model 1^a^	Model 2^b^	Model 3^c^
	PRR (95% CI)	PRR (95% CI)	PRR (95% CI)
Age Category			
55–59	0.84 (0.58, 1.22)	-	0.78 (0.51, 1.21)
60–64	0.86 (0.60, 1.23)	-	0.71 (0.47, 1.08)
65–74	0.64 (0.46, 0.90)^**^	-	0.56 (0.38, 0.83)^**^
≥75	Reference	-	Reference
Female (vs. Male)	1.01 (0.79, 1.28)	-	1.08 (0.82, 1.44)
Health Conditions (Yes vs. No)			
Distress	2.53 (1.58, 4.04)^***^	2.46 (1.54, 3.91)^***^	2.25 (1.31, 3.87)^**^
Head Injury	1.99 (1.37, 2.89)^***^	1.90 (1.31, 2.77)^***^	1.58 (1.00, 2.51)^*^
Alcohol Use Disorder	1.78 (1.24, 2.57)^**^	1.79 (1.24, 2.59)^**^	1.24 (0.77, 2.01)
Diabetes	1.40 (1.11, 1.76)^**^	1.48 (1.30, 1.68)^***^	1.59 (1.21, 2.10)^***^
Hypertension	1.27 (1.00, 1.63)^*^	1.27 (1.11, 1.45)^***^	1.02 (0.76, 1.36)
Stroke	1.29 (0.81, 2.07)	1.27 (0.80, 2.02)	1.18 (0.70, 1.99)
Heart Disease	1.26 (0.93, 1.70)	1.30 (0.95, 1.76)	0.96 (0.67, 1.38)
Obesity	1.25 (0.97, 1.59)	1.23 (0.96, 1.57)	0.87 (0.64, 1.16)
# of health conditions	1.18 (1.09, 1.27)^***^	1.18 (1.09, 1.27)^***^	-

*PRR*: prevalene rate ratio, *CI* confidence interval.

^a^Model 1: unadjusted model.

^b^Model 2: only adjusted for age and sex.

^c^Model 3: adjusted for age, sex and all other health conditions.

^*^p < 0.05, ^**^p < 0.01, ^***^p < 0.001.

**Fig. 1:**
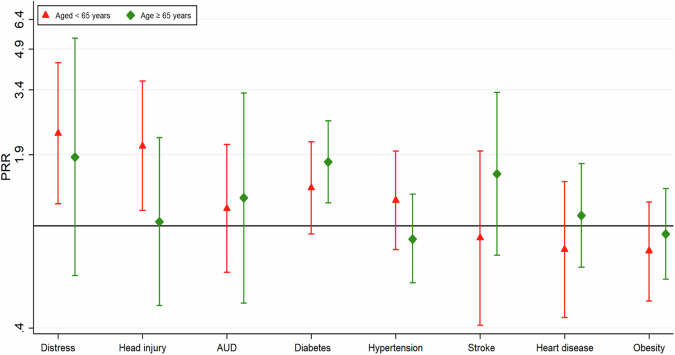
This fi gure illustrates the prevalence rate ratio between health conditions and AD8 scores among American Indian(AI) aged <65 years versus those aged ≥ 65 years. Association Between Health Conditions and AD8 Scores in American Indian Participants Stratified by Age Groups using Negative Binomial Regression in the Fully Adjusted Model (Model 3 in Table 2). *Note:* Model adjusted for age, sex and all other health conditions. Interaction terms between age groups and these health conditions are not significant (*p* > 0.05). Abbreviations: PRR prevalence rate ratio, CI confidence interval.

Figure 1 and supplemental Table 1 present the associations between these health conditions and AD8 scores among younger (<65 years) and older (≥65 years) AI adults. Stratified analyses by age group revealed that among younger adults, distress and head injury were associated with 130% and 105% higher AD8 score (i.e. worse cognitive function), respectively (PRR = 2.30 and 2.05); both associations were statistically significant. However, diabetes (PRR = 1.78) was significantly associated with worse cognitive performance only in the older group. We tested interactions between health conditions and age group; yet, none of the interaction terms were statistically significant (all *p* > 0.05).

The associations between health conditions and AD8 scores stratified by sex were shown in Supplemental Table 2. In the fully adjusted model (Model 3), age was differentially associated with AD8 scores in males and females. Among AI males, those aged 55–59 years had a 4.72-fold higher AD8 score (i.e., worse cognitive function) compared with those aged ≥75 years. In contrast, among females, younger age was significantly associated with lower AD8 score (i.e., better cognitive performance), with PRRs of 0.62 for ages 60–64 and 0.46 for ages 65–74, compared with those aged ≥75 years. Additionally, distress (PRR = 2.32) showed a significant association with worse cognitive performance only among females, while head injury (PRR = 2.89) was significantly associated with worse cognitive performance only among males.

## Discussion

Our study revealed a high prevalence of cognitive impairment among these AI adults, as assessed by the adapted AD8, with an unexpected, elevated percentages observed in younger respondents. Distress and head injury were significantly associated with increased AD8 scores among relatively younger adults (<65 years), whereas diabetes was significantly associated with higher AD8 scores in the older group (≥65 years). With respect to sex differences, distress was significantly associated with higher AD8 scores only among females, whereas head injury was significantly associated with higher AD8 scores only among males.

Although the age distribution in our sample reflects a slightly lower proportion of individuals aged 55–64 years compared to the 2023 Census data (45.6% vs. 50.5%)^9^, our study reveals a higher proportion of cognitive impairment among younger AI individuals, with 38.2% affected among those aged 55–64 years. This finding aligns with prior studies reporting that AI/AN younger adults (45–64 years) had a higher prevalence of subjective cognitive decline than older (>65 years) AI/AN participants and other racial/ethnic groups in the same age range, based on Behavioral Risk Factor Surveillance System (BRFSS) data from 2015 to 2020^10,11^. This trend may be attributed to the elevated prevalence of risks such as head injury among younger AIs, driven by socio-economic challenges and higher rates of accidents and violence. Notably, in contrast to previous research, our study evidenced a higher proportion of cognitive impairment among younger AI adults [38.2% in our study vs. 18.6% in previous studies using BRFSS data^10,11^]. However, prior studies using BRFSS data defined subjective cognitive decline based on a single question about cognitive function, whereas we used the adapted AD8 questionnaire, classifying individuals as cognitively impaired if they answered “Yes” to at least two of eight cognitive health-related questions. Our younger adults include individuals 55–64 years, while the previous studies have considered a broader age range of 45–64 years. These variations may account for the higher prevalence observed in our study.

It is well recognized that emotional distress is associated with worse cognitive functions^12^. A recent systematic review reported that approximately 82% of the 22 reviewed articles demonstrated an association between emotional distress and an increased risk of dementia among adults aged 38 to 88 years^12^. These findings align with our results: emotional distress is associated with an increased risk of worse cognitive performance. Several explanations may account for this association. The 2024 *Lancet* report highlighted that several modifiable risk factors are linked to ADRD^6^. Distress has been associated with several of these risk factors^13^, notably depression, smoking, and hypertension, which may contribute to the pathway between distress and cognitive impairment. Additionally, emotional distress has been found to be associated with changes in the hippocampus, potentially mediated by elevated levels of glucocorticoids, which may have detrimental effects on cognitive health^14^. Moreover, such distress is considered a contributing factor to the progression of Alzheimer’s disease, linked to its pathological hallmarks. It is also associated with diminished levels of brain-derived neurotrophic factor, which is thought to protect against ADRD^15^. These connections suggest a potential biological mechanism that may exacerbate neurodegenerative issues.

We observed a statistically significant association between distress and worse cognitive performance, with stronger associations among younger AI adults than older adults, and among females compared with males. Cultural factors likely contribute to stress and mental health issues, potentially exacerbating cognitive symptoms even at younger ages^16^. In addition, because the AD8 relies on self-reported information, higher scores among younger individuals may reflect heightened awareness of or sensitivity to cognitive changes in the context of complex health and social challenges. Furthermore, previous research has reported an association between emotional distress and lower levels of social engagement^17^, and our prior study found that lower social engagement was associated with a higher risk of cognitive impairment among AI/AN females, but not among AI/AN males^18^ Together, these factors may help explain the significant association between distress and worse cognitive performance observed among younger adults and among females in our sample.

Our findings are consistent with recent research that underscored a significant association between diabetes and cognitive impairment, particularly within the AI population, which bears the highest burden of diabetes across all ages in the U.S.^19,20^. Initially categorized as a late-life risk factor for dementia in the 2020 *Lancet Commission* report^21^, diabetes has been reclassified as a mid-life risk factor in the 2024 update^6^, reflecting its potential impact across the life span. Although the association between diabetes and AD8 scores was statistically significant only among older adults, the magnitude of the association among younger participants was only slightly lower, suggesting the lack of significance may be due to relatively smaller sample size in this age group. The higher prevalence of diabetes among older adults may also partially explain the observed age-specific significance. Additionally, a meta-analysis encompassing over 2.3 million individuals with type 2 diabetes from 14 cohort studies, including 102,174 dementia cases, found that diabetes was associated with 60% higher risk of any form of dementia^22^. Another pooled analysis involving over 48,000 individuals from 15 countries indicates that diabetes is independently associated with poorer cognitive performance and more rapid cognitive decline^23^. In the Strong Heart Study, the largest longitudinal studies of cardiovascular disease among older AI populations, type 2 diabetes was linked to reduced performance on measures of executive function, including verbal fluency, processing speed, and working memory^24^.

Diabetes contributes to cognitive impairment through chronic hyperglycemia and its adverse effects on the brain’s vascular system. Chronic hyperglycemia accelerates the production of advanced glycation end-products, which accumulate in the blood and tissues, potentially leading to neuronal degeneration and vascular endothelial damage, thus impairing cognitive function^25^. Vascular brain injury, in its various forms, is closely linked to cognitive decline and dementia^26^.

Our findings indicate that the history of head injury is significantly associated with increased risk of cognitive impairment among relatively younger AI individuals. TBI is a major concern within this population^27^. TBI is defined as an alteration in brain function or evidence of brain pathology resulting from an external force. The severity of TBI can lead to a broad spectrum of functional changes, which may include impairments in cognition (e.g., memory and reasoning), sensation (e.g., vision and balance), language (e.g., communication and comprehension), and emotion (e.g., depression, personality changes, and inappropriate social behavior). Research has consistently shown that AIs experience higher rates of TBI compared to other racial/ethnic groups^28^. For instance, AI adults had the highest age-adjusted rates of TBI-related deaths in the U.S., with a rate of 28.3 per 100,000, compared to 19.4, 16.6, 11.3, and 8.0 per 100,000 for non-Hispanic Whites, non-Hispanic Blacks, Hispanics, and non-Hispanic Asians and Pacific Islanders, respectively^28^. Additionally, another study highlighted that younger AI individuals aged 18–34 years had the highest rate of TBI-related emergency department visits^27^. According to the 2014 CDC report, AI/AN adults aged 15 to 34 years had the highest rates of TBI-related hospitalizations and deaths due to motor vehicle crashes^29^. The Indian Health Service reports that AI/AN peoples have a motor vehicle-related death rate that is 200% higher than other racial/ethnic groups^30^. These early life exposures to head injury may have a profound impact on brain function and potentially contribute to the early onset of cognitive impairment. In addition to accidents, historical traumas such as forced relocation and boarding school attendance also contribute to the persistence of intentional injuries, including head injuries, within AI communities^31^. Furthermore, disparities in access to healthcare services following TBI may exacerbate adverse outcomes, including cognitive impairment. AI adults are less likely than non-Hispanic Whites to be discharged to facilities with additional health services, such as inpatient rehabilitation or long-term care, even after adjusting for injury severity^32^.

Young adults with a history of head injury are more likely to exhibit impairments in cognitive function. Evidence from both community-based studies^33^ of healthy young adults and clinical studies of young adult populations^34^ indicates that self-reported head injury is associated with increased cognitive impulsivity. Furthermore, neuropsychological performance among survivors of TBI is significantly lower than that of neurotypical controls, as assessed by theoretically derived composite measures of verbal and visual memory, executive function, attention, and cognitive reserve in young adults^35^. Injury-related characteristics, including level of consciousness and Glasgow Coma Scale scores, have been shown to be significantly associated with deficits in divided attention and memory function (*p* < 0.05), respectively, in cohorts of individuals aged 18–59 years with mild to moderate TBI^36^. Future research should focus on strategies to reduce head injuries and enhance post-injury care among young AI adults. Furthermore, with respect to sex differences in head injury and cognitive impairment, we found that the association between head injury and worse cognitive impairment was stronger among AI males than females. In our study sample, males had a slightly higher prevalence of head injury than females (11.5% vs. 10.4%). Moreover, a recent study reported that males had higher incidence rates of TBI than females for nearly all causes, as well as for minor, moderate, and severe levels of head injury^37^. These factors may contribute to the stronger association observed in males compared with females in our study.

Although stroke was not significantly associated with AD8 scores in either younger or older age group, the estimated associations differed substantially in magnitude and direction between younger and older adults. A recent scoping review of randomized clinical trials and epidemiological studies suggests that cognitive impairment within the first year after a stroke is highly prevalent, ranging from mild to severe^38^. Aging is a robust risk factor for stroke, with approximately 75% of all strokes occurring among those aged 65 years and older^39^. Several potential mechanisms have been proposed for the association between stroke and cognitive impairment among older adults. Neurodegenerative processes, like impaired amyloid clearance or activation of autoimmune responses against brain antigens, have been suggested to be associated with stroke^40^. Additionally, extensive alterations in white matter associated with stroke might elevate the risk of memory decline and contribute to cortical gray matter atrophy, thereby increasing the risk of cognitive problems^41^.

Furthermore, cognitive deficits are observed in more than 70% of stroke survivors^42^, and advancing age is consistently associated with accelerated cognitive decline following stroke. For instance, findings from the REGARDS (Reasons for Geographic and Racial Differences in Stroke) study demonstrated that each one-year increase in baseline age was associated with a 17% higher odds of developing cognitive impairment per year of follow-up^43^. In addition, stroke survivors with lower educational attainment were 1.8 times more likely to develop poststroke dementia^44^. Evidence from studies of older adults further indicates that the prevalence ratio of poststroke cognitive impairment increases by approximately 2% for each additional year of age^45^. Moreover, a prospective observational cohort study of patients with spontaneous intracerebral hemorrhage reported that each 10-year increase in age was associated with a 34% higher risk of incident dementia^46^. The evidence supports our finding that stroke is more strongly associated with cognitive impairment in older AI adults compared with younger adults, likely reflecting the compounded effects of age-related vulnerability and poststroke cognitive decline.

While health conditions such as cardiovascular disease and obesity have strong links to cognitive decline in the general population, our study suggests these conditions may exhibit a more limited association with an increased risk of cognitive impairment among AI adults. One potential explanation is the high prevalence of diabetes in the AI community, which may overshadow the cognitive effects of other related conditions and make them less prominent contributor to cognitive impairment. Additionally, the concordance between self-reported and claim-based administrative data is moderate for heart disease and socio-economic factors may affect recognition and reporting of these health conditions^47^. Consequently, the validity of self-reported measures for heart disease and obesity may be limited. The potential measurement errors for these health conditions may have contributed to the weaker associations between heart disease, obesity, and cognitive impairment found in our study.

Our study possesses several strengths and limitations. To our knowledge, this is the first investigation to assess the proportion of cognitive impairment among different age groups across multiple sites within the AI communities in the U.S. using the AD8. Unexpectedly, 40.3% of respondents aged 55–59 years met the AD8 criteria for cognitive impairment, underscoring the need for early detection in this population. Furthermore, emotional distress and diabetes as the lifelong key factors associated with cognitive impairment, head injury as the significant risk factors associated with early-life cognitive impairment, and stroke as a late-life risk factor: findings that can guide age-specific prevention strategies to mitigate the high burden of cognitive impairment among AI adults.

However, several limitations should be considered. First, our study sample was one of convenience, which may not adequately represent AIs at large, thereby limiting the generalizability of these findings. Second, the convenience sampling method and volunteering basis of these surveys likely attracted those with poorer health status, especially among the relatively younger participants of this study, potentially leading to a higher observed prevalence of cognitive impairment and age-related associations with AD8 scores that differed by sex. Third, health conditions were self-reported without verification by medical records, potentially introducing recall bias and possibly affecting the accuracy of the associations between cognitive impairment and health conditions. Next, although prior studies have applied the AD8 among younger adults and demonstrated that it is an easy-to-administer and valid screening tool for detecting early cognitive impairment^48,49^, several items assess independence in activities of daily living, which typically reflect later-stage impairment in older population. These characteristics of the instrument may reduce its specificity when applied to younger adults. Thus, as the AD8 was originally developed for older populations (≥65 years), further validation of its applicability to younger age groups is warranted in future studies. Additionally, the AD8 scores relied on participants’ self-perception of cognitive changes without objective clinical evaluations. Despite this, a systematic review supports the value of the AD8 as a screener for cognitive impairment, particularly in primary care settings^50^. Furthermore, the survey was conducted in English only, which may have limited participation among AI individuals who primarily speak Indigenous languages or have limited English proficiency. Lastly, since our study employed a cross-sectional design, it cannot establish the temporality or causality among health conditions and cognitive impairment.

In conclusion, our study reveals a high prevalence of cognitive impairment among relatively younger AI adults, in which emotional distress, head injury, and diabetes were significantly associated with increased risk of cognitive impairment. The adapted AD8 questionnaire appears to be a convenient and feasible tool for identifying cognitive impairment among AI adults and could be recommended for both middle-aged and older adults. Future efforts to develop culturally tailored intervention programs aimed at addressing the potential risk factors identified in this study may have great potential to reduce the heavy burden of cognitive impairment in this population.

## Method

### Study population

This study was organized by the Centers for American Indian and Alaska Native (AI/AN) Health (CAIANH) and implemented through three Satellite Centers: Pacific Northwest Satellite Center (Seattle, WA), Rocky Mountain Satellite Center (Denver, CO), and Northern Plains Satellite Center (Minneapolis, MN). We conducted an anonymous, cross-sectional survey among a convenience sample of urban and rural AI adults residing near the three geographic centers. This approach ensured local community stakeholder involvement in project planning and inclusion of regionally appropriate elements for implementation. Our inclusion criteria included those with self-reported AI/AN people aged 55 years or older. The 712 respondents were recruited through community events and email outreach. Participants were compensated $20 for completing the survey.

### Ethics approval and informed consent

The Institutional Review Boards (IRBs) of the universities hosting these Satellite Centers reviewed and approved the human subject protection measures. This study was reviewed and approved by the Colorado Multiple IRBs (COMIRB; FWA A00005070). This project was determined to involve minimal risk and was granted a waiver of consent. The survey was de-identified; therefore, the only record linking participants to the project would have been the consent form, which would have created an unnecessary and undue risk of a data breach. Participants were provided with an information sheet describing the study that did not require a signature. The study team reviewed the information sheet with them prior to survey participation.

Respondents were asked to complete a pencil-and-paper format, self-report survey comprised of an adapted version of the AD8, select chronic health conditions, and sociodemographic questions. The entire survey required approximately 15 min to complete.

### Outcome measurement: AD8 and its adaptation

The AD8 is a commonly used measure developed and validated through longitudinal studies of memory and aging to assess the presence of dementia^50^. It was originally based on the Clinical Dementia Rating (CDR), a well-established informant-based scale widely utilized in clinical research to aid in the diagnosis of dementia^8^. The AD8 has been subsequently validated for self-administration^51^, making it particularly useful in contexts where reliable informants are unavailable or when there are limited caregiver resources in clinical settings. The AD8 questionnaire is comprised of 8 items which the respondent endorses as Yes (1) or No (0) based on their perceptions over the last “several years”. The eight questions ask about: (1) problems with judgment (e.g., problems making decisions, bad financial decisions, problems with thinking); (2) less interest in hobbies/activities; (3) repeats the same things over and over (questions, stories, or statements); (4) trouble learning how to use a tool, appliance, or gadget (e.g., VCR, computer, microwave, remote control); (5) forgets the correct month or year; (6) trouble handling complicated financial affairs (e.g., balancing checkbook, income taxes, paying bills); (7) trouble remembering appointments; and 8) daily problems with thinking and/or memory. Item-level scores are summed, resulting in a total score ranging from 0 to 8 which was the primary outcome of the present study. Higher total AD8 scores are indicative of a greater likelihood of cognitive impairment and worse cognitive function. The suggested cut-off score for dementia or cognitive impairment is 2 or greater^52^. The AD8 is highly correlated with gold standard evaluations including the CDR14, neuropsychological testing^53^, and imaging and cerebrospinal fluid biomarkers of AD^54^. A recent systematic review and meta-analysis reported that the sensitivity and specificity of the self-administered AD8 for detecting dementia were 82% and 75%, respectively^50^.

The use of the AD8 among members of nearly 20 different nations revealed remarkably consistent psychometric properties and factor structure^7,50^. However, our prior work suggested that the reliability of measures of cognitive status and functioning can be constrained by implicit cultural assumptions that compromise their performance among AI respondents whose backgrounds differ substantially from those of the original referent populations^55–57^. Ignoring how such assumptions may negatively affect comprehension and acceptability can contribute to a host of problems ranging from inconsistent answers and missing data to routinized responses that bear little relationship to lived experience^58^. Therefore, we chose to first examine such features as we prepared to assess risk of dementia among community dwelling members of a geographically and culturally diverse array of older AI adults.

An initial review of the AD8 by our Community Advisory Board (CAB), comprised of AI/AN advocates and family members as well as providers, immediately suggested that various aspects of it warranted closer attention. Specifically, they encouraged modifications of the instructional set, response labels, item word choice, grammatical structure, and examples to optimize older AI/AN peoples’ understanding, perceived relevance, and acceptance of the measure. We therefore undertook a careful, systematic adaptation of the AD8 along these lines, employing multiple iterative exchanges with CAB members, which began with a global discussion of the purpose of the AD8, and then solicited individual and group recommendations, reactions, and further revisions to item content and form.

The original AD8 (left) and final version (right) adapted for our use was presented side-by-side in supplemental Fig. 1. We adopted a conservative approach throughout, constantly examining whether a recommendation represented a potential change in the meaning of an item, which we studiously avoided. Turning first to the instructional set, our advisers recommended greater clarity about the expectation for answering each question, by noting both “Yes” and “No” options and linking each more specifically to the wording of the response alternatives. They also strongly argued the word “cognitive”, given many older AI/AN adults’ lack of familiarity with this word, was less likely to be understood than “thinking and memory” which are offered as alternatives in the original version. Hence, to avoid initial confusion, they recommended simply deleting reference to “cognitive” [and substituting “thinking and memory”]. Moreover, CAB members asserted that the task of associating “a change” with “problems” is a more complex task than might first appear, and the putative mechanism of action too general. Instead, they proposed a more direct reference to “trouble with thinking and memory” as the cause for said change. Then, too, our advisers suggested that positioning the timeframe of “last several years” between “a change” and “cognitive problems” was distracting and interrupted the presumed connection, leading to their recommendation to place it at the end of the sentence. Lastly, several CAB members pointed out that asking the respondent to “Remember …” was especially ironic as part of the instruction for completing a series of questions about the consequences of problems with thinking and memory. Thus, we removed the word “Remember” from the question.

To increase respondent comprehension, several revisions in word choice, substitution, and/or placement were made consistently across items. For example, broad terms like “judgment” (Item 1), “hobbies” (Item 2), “same things” (Item 3), and “financial affairs” (Item 6) were deleted and the original illustrations accepted. Our advisers also encouraged greater specificity, e.g., substituting “focusing one’s thoughts” for “problems thinking” (Item 1), and “crafts and cultural activities” for hobbies/activities” (Item 2). They repeatedly emphasized unadorned language, substituting “money decisions” for “financial decisions” (Item 6), and avoiding terms such as “affairs” (Item 6). Placement of the timeframe arose again as a matter of concern. With respect to Item 8, CAB members pointed out that positioning frequency of occurrence at before asking about “thinking and/or memory problems” emphasized time over the focal problem. They felt first stating the concern and then asking about occurrence was more consistent with rules of discourse in their communities.

As is standard, we subsequently calculated the total AD8 score for each individual as the total numbers of “Yes” responses for AD8 questionnaires (ranging from 0 to 8). Individuals with higher total AD8 scores are more likely to have cognitive impairment or poorer cognitive function. Based on the suggested cut-off score for identifying dementia or cognitive impairment (2 or greater)^52^, individuals were identified as having cognitive impairment if their AD8 score was 2 or more, while those with an AD8 score of less than 2 were classified as not having cognitive impairment.

### Health conditions and demographic characteristics

Health conditions were identified based on self-reported medical history using the following questions: “Have you ever been told by a health care provider that you have any of the following conditions? (1) diabetes; (2) hypertension; (3) heart disease; (4) stroke; (5) head injury; (6) alcohol use disorder (AUD); and (7) obesity.” Additionally, the 6-item Kessler Psychological Distress Scale (K6 scale), which has demonstrated outstanding internal consistency and reliability with a Cronbach’s alpha of 0.89, was employed to assess the frequency of non-specific psychological distress^59^. Specifically, participants rated how often they experienced various feelings during the past 30 days, including nervousness, hopelessness, restlessness/fidgetiness, depression that nothing could cheer up, everything being an effort, and worthlessness. Responses were recorded on the following scale: “0-None of the time”, “1-A little of the time”, “2-Some of the time”, “3-Most of the time”, and “4-All of the time”. The mean score across these six items, ranging from 0 to 4 (with the total scores ranging from 0 to 24), was calculated, with higher scores indicating greater mental distress. In this study, distress was defined as having a K6 mean score of 2.17 or higher (i.e., 13 divided by 6). The score of 13 represents the established thresholds used in prior research to identify individuals experiencing severe mental distress^60^. All health conditions, including distress, were defined as binary variables, recorded as “Yes” if there was a history of the condition and “No” otherwise. A health condition index was constructed to reflect the total number of comorbid conditions reported by each participant.

### Statistical analysis

Demographic and clinical characteristics were summarized using frequency distributions for categorical variables and mean and standard deviation for continuous variables across the overall study sample. These characteristics were further stratified by cognitive impairment status (AD8 score of 0–1 vs. ≥2) and age group (<65 vs. ≥65 years). To compare statistics across these different stratifications, two-sample t-tests (or ANOVA for three or more groups) were used for continuous variables, while chi-square tests were employed for categorical variables.

Subsequently, we performed negative binomial regression to evaluate the associations between health conditions and the total AD8 scores. The initial adjusted model included a single health condition together with age and sex, whereas the fully adjusted model further incorporated all other health conditions. Prevalence rate ratios (PRRs) and corresponding 95% confidence intervals (CIs) from negative binomial regression models were reported. All statistical analyses were conducted using SAS 9.4.

## Supplementary information

Supplementary Information

## Data Availability

The study data have not been made available for sharing broadly because of Tribal regulations regarding data confidentiality and security. However, we are happy to provide the SAS codes upon request. The research center where Dr. Spero M. Manson, one of the multiple principal investigators (MPIs), works has developed a process to explore opportunities to share project data that would benefit the health of the AI people engaged in the project.
