# Supplementary material for: Cognitive impairment and its associated health conditions in American Indian communities
Source: NPJ Dement. 2026 Apr 29;2(1):34. doi: 10.1038/s44400-026-00080-0 (PMC13128437; doi:10.1038/s44400-026-00080-0)
Supplement: Supplementary file 1 — Supplementary Information [file 44400_2026_80_MOESM1_ESM.docx]

**Supplementary Online Content**

**Supplemental Figure 1.** The original AD8 (left) and final version (right) adapted AD8 used in the study

**Supplemental Table 1.** Association Between Health Conditions and AD8 Scores in American Indian Adults Stratified by Age using Negative Binomial Regression in the Fully Adjusted Model (Model 3 in Table 2)

**Supplemental Table 2.** Association Between Risk Factors and AD8 scores in American Indian Participants Using Negative Binomial Regression, stratified by sex.

**Supplemental Figure 1.** The original AD8 (left) and final version (right) adapted AD8 used in the study

**
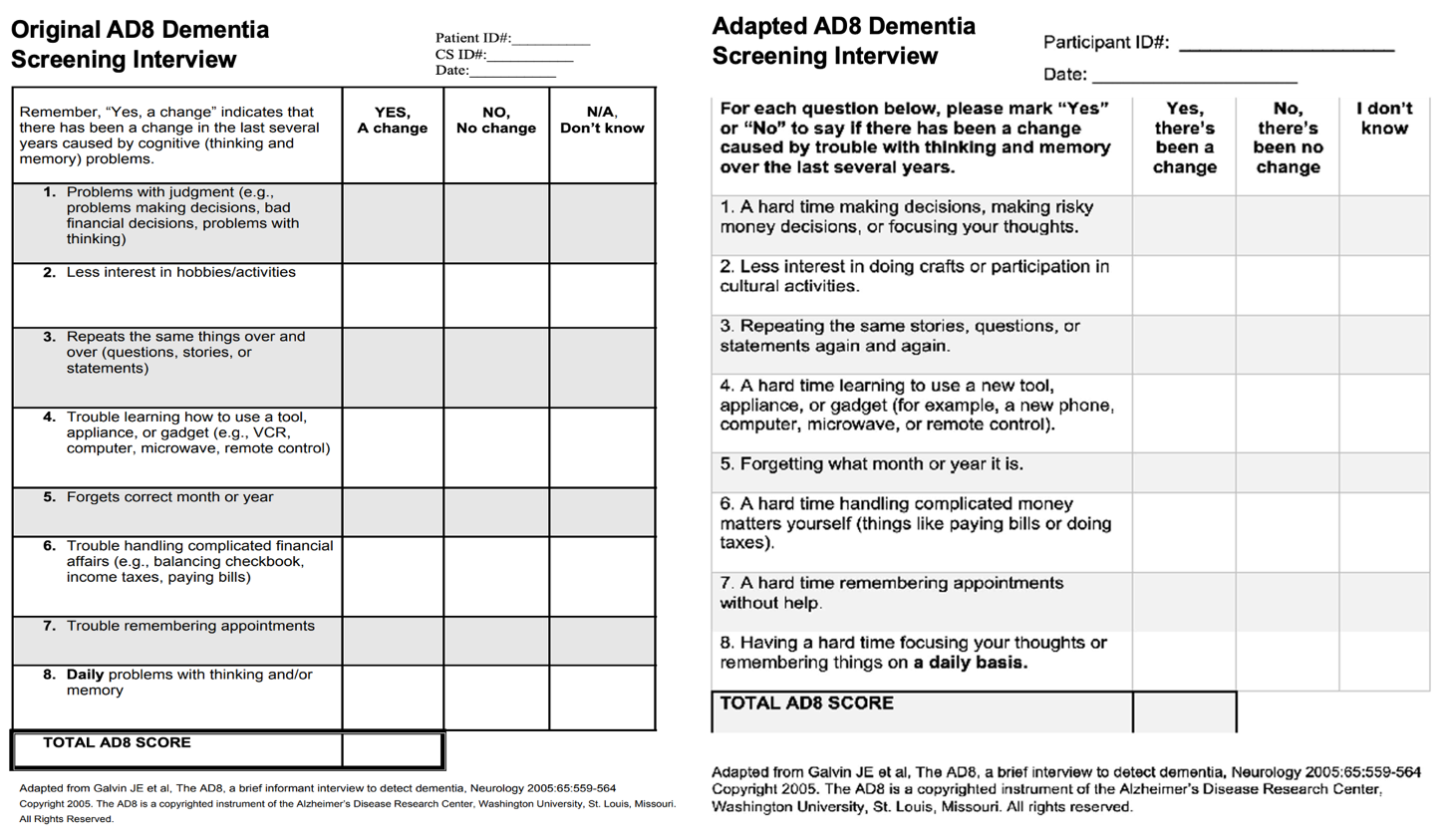
**

**Supplemental Table 1.** Association Between Health Conditions and AD8 Scores in American Indian Adults Stratified by Age using Negative Binomial Regression in the Fully Adjusted Model (Model 3 in Table 2)

|  | **Aged < 65 years** | **Aged ≥ 65 years** | **P-value (Interaction with Age Groups)** |
| --- | --- | --- | --- |
|  | **PRR (95% CI)** | **PRR (95% CI)** | **PRR (95% CI)** |
| **Health Conditions (Yes vs. No)** |  |  |  |
| **Distress** | **2.30 (1.22-4.33)^*^** | 1.86 (0.64-5.40) | 0.930 |
| **Head Injury** | **2.05 (1.15-3.68)^*^** | 1.04 (0.49-2.21) | 0.251 |
| **Alcohol Use Disorder** | 1.17 (0.66-2.08) | 1.29 (0.50-3.30) | 0.641 |
| **Diabetes** | 1.41 (0.93-2.13) | **1.78 (1.23-2.57)^**^** | 0.412 |
| **Hypertension** | 1.26 (0.81-1.96) | 0.89 (0.60-1.33) | 0.743 |
| **Stroke** | 0.90 (0.41-1.96) | 1.60 (0.77-3.32) | 0.266 |
| **Heart Disease** | 0.81 (0.44-1.49) | 1.10 (0.69-1.75) | 0.354 |
| **Obesity** | 0.80 (0.51-1.24) | 0.93 (0.62-1.40) | 0.635 |

*Note:* Model adjusted for age, sex and all other health conditions.

Abbreviations: PRR: prevalence rate ratio; CI: confidence interval.

^*^p<0.05, ^**^p<0.01, ^***^p<0.001

**Supplemental Table 2.** Association Between Risk Factors and AD8 scores in American Indian Participants Using Negative Binomial Regression, stratified by sex.

|  | **Model 1^a^** | | **Model 2^b^** | | **Model 3^c^** | |
| --- | --- | --- | --- | --- | --- | --- |
|  | **Male (n=211)** | **Female (n=501)** | **Male (n=211)** | **Female (n=501)** | **Male (n=211)** | **Female (n=501)** |
|  | **PRR (95% CI)** | **PRR (95% CI)** | **PRR (95% CI)** | **PRR (95% CI)** | **PRR (95% CI)** | **PRR (95% CI)** |
| **Age Category** |  |  |  |  |  |  |
| 55-59 | 1.12 (0.48, 2.65) | 0.81 (0.53, 1.22) | - | - | **4.72 (1.20, 18.54)^*^** | 0.66 (0.41, 1.05) |
| 60-64 | 1.28 (0.56, 2.89) | 0.78 (0.52, 1.17) | - | - | 3.31 (0.91, 12.05) | **0.62 (0.40, 0.96)^*^** |
| 65-74 | 1.00 (0.45, 2.22) | **0.57 (0.39, 0.83)^**^** | - | - | 2.88 (0.79, 10.44) | **0.46 (0.30, 0.69)^***^** |
| ≥75 | Reference | Reference | - | - | Reference | Reference |
| **Health Conditions (Yes vs. No)** |  |  |  |  |  |  |
| Distress | **2.50 (1.00, 6.21)^*^** | **2.54 (1.47, 4.39)^***^** | 2.39 (0.95, 6.01) | **2.46 (1.44, 4.20)^**^** | 2.12 (0.66, 6.77) | **2.32 (1.26, 4.27)^**^** |
| Head Injury | **2.78 (1.38, 5.59)^**^** | **1.71 (1.10, 2.65)^*^** | **3.30 (1.53, 7.09)^**^** | **1.66 (1.07, 2.58)^*^** | **2.89 (1.16, 7.18)^*^** | 1.31 (0.76, 2.27) |
| Alcohol Use Disorder | **2.11 (1.20, 3.73)^**^** | 1.62 (0.98, 2.66) | **2.33 (1.27, 4.24)^**^** | **1.70 (1.04, 2.77)^*^** | 0.93 (0.42, 2.05) | 1.48 (0.79, 2.78) |
| Diabetes | **1.58 (1.01, 2.48)^*^** | **1.33 (1.02, 1.73)^*^** | **1.75 (1.36, 2.24)^***^** | **1.40 (1.20, 1.63)^***^** | **1.87 (1.01, 3.46)^*^** | **1.54 (1.13, 2.09)^**^** |
| Hypertension | 1.53 (0.94, 2.50) | 1.18 (0.89, 1.57) | **1.61 (1.25, 2.07)^***^** | **1.18 (1.01, 1.39)^*^** | 0.89 (0.44, 1.77) | 1.03 (0.75, 1.43) |
| Stroke | 1.44 (0.64, 3.25) | 1.22 (0.68, 2.19) | 1.58 (0.68, 3.65) | 1.17 (0.66, 2.09) | 1.34 (0.55, 3.30) | 1.16 (0.60, 2.26) |
| Heart Disease | 1.41 (0.83, 2.40) | 1.21 (0.83, 1.76) | 1.73 (0.97, 3.10) | 1.17 (0.81, 1.70) | 1.25 (0.63, 2.47) | 0.81 (0.53, 1.26) |
| Obesity | 1.39 (0.81, 2.36) | 1.19 (0.90, 1.58) | 1.49 (0.85, 2.60) | 1.19 (0.90, 1.57) | 1.11 (0.56, 2.19) | 0.84 (0.61, 1.17) |
| # of health conditions | **1.22 (1.07, 1.40)^**^** | **1.16 (1.05, 1.27)^**^** | **1.26 (1.09, 1.45)^**^** | **1.15 (1.05, 1.26)^**^** | - | - |

^a^Model 1: unadjusted model.

^b^Model 2: only adjusted for age.

^c^Model 3: adjusted for age and all health conditions.

Abbreviations: PRR: prevalence rate ratio; CI: confidence interval.

*p<0.05, **p<0.01, ***p<0.001
